# Pilot Study on Acute Effects of Pharmacological Intraperitoneal L-Homoarginine on Homeostasis of Lysine and Other Amino Acids in a Rat Model of Isoprenaline-Induced Takotsubo Cardiomyopathy

**DOI:** 10.3390/ijms23094734

**Published:** 2022-04-25

**Authors:** Dimitrios Tsikas, Björn Redfors

**Affiliations:** 1 Institute of Toxicology, Core Unit Proteomics, Hannover Medical School, 30623 Hannover, Germany; 2 Department of Cardiology, Sahlgrenska University Hospital, 413 45 Gothenburg, Sweden; bjoern.redfors@wlab.gu.se

**Keywords:** AGAT, amino acids, L-arginine, GC-MS, L-homoarginine, isoprenaline, L-lysine, polyamines, putrescine, spermidine, sarcosine

## Abstract

L-Arginine:glycine amidinotransferase (AGAT) catalyzes the formation of L-homoarginine (hArg) and L-ornithine (Orn) from L-arginine (Arg) and L-lysine (Lys): Arg + Lys ↔ hArg + Orn; equilibrium constant *K*_hArg_. AGAT also catalyzes the formation of guanidinoacetate (GAA) and Orn from Arg and glycine (Gly): Arg + Gly ↔ GAA + Orn; equilibrium constant *K*_GAA_. In humans, pharmacological hArg is metabolized to Lys. Low circulating and low excretory concentrations of hArg are associated with worse outcomes and mortality in the renal and cardiovascular systems. The metabolism and pharmacology of hArg have been little investigated. In the present study, we investigated the effects of pharmacological hArg (i.p., 0, 20, 220, 440 mg/kg at time point 0 min) on amino acids homeostasis in a rat model of isoprenaline-induced takotsubo cardiomyopathy (i.p., 50 mg/kg at time point 15 min). We measured by gas chromatography-mass spectrometry free and proteinic amino acids, as well as the polyamines putrescine and spermidine in the heart, lung, kidney, and liver of ten rats sacrificed at various time points (range, 0 to 126 min). hArg administration resulted in multiple changes in the tissue contents of several free and proteinic amino acids, as well as in the putrescine-spermidine molar ratio, an indicator of polyamines catabolism. Our results suggest that Lys and Arg are major metabolites of pharmacological hArg. Kidneys and heart seem to play a major metabolic role for hArg. Circulating Lys does not change over time, yet there is a considerable interchange of free Lys between organs, notably kidney and heart, during the presence of isoprenaline in the rats (time range, 15 to 90 min). Antidromic changes were observed for *K*_hArg_ and *K*_GAA_, notably in the heart in this time window. Our study shows for the first time that free hArg and sarcosine (*N*-methylglycine) are positively associated with each other. The acute effects of high-dosed hArg administration and isoprenaline on various amino acids and on AGAT-catalyzed reaction in the heart, lung, kidney, and liver are detailed and discussed.

## 1. Introduction

**Scheme 1:**
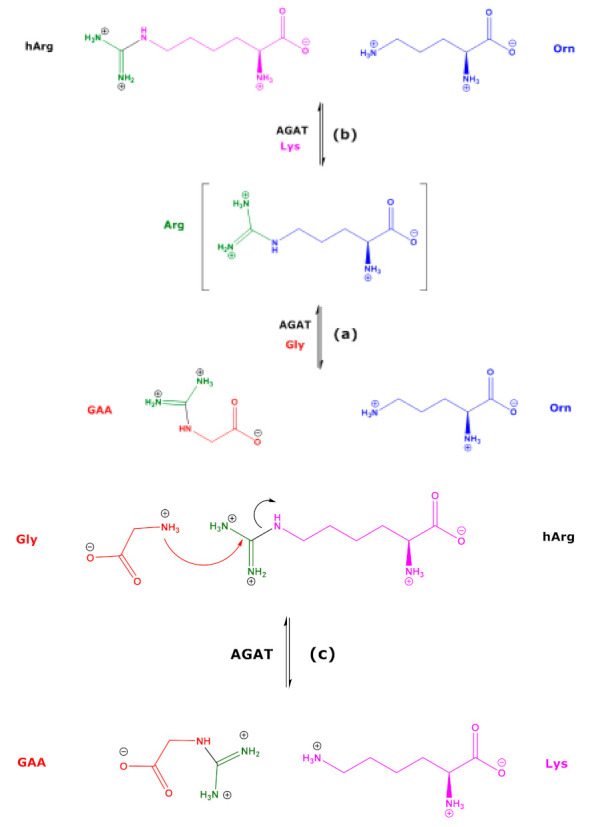
Simplified schematic of the AGAT-catalyzed synthesis of guanidinoacetate (GAA) from L-arginine (Arg) and glycine (Gly) (**a**), of L-homoarginine (hArg) from L-arginine and L-lysine (Lys) (**b**), and of the AGAT-catalyzed synthesis of GAA from L-homoarginine and Gly (**c**).

L-Arginine:glycine amidinotransferase (AGAT), also known as glycine amidinotransferase (GATM; EC 2.1.4.1), catalyzes the formation of guanidinoacetate (GAA) from L-arginine (Arg) and glycine (Gly) with L-ornithine (Orn) being the second reaction product (reaction (a) in Scheme 1) [1]. The catalytic process includes a nucleophilic attack of the sulfhydryl (SH) of a cysteine (Cys) moiety of AGAT on the guanidino C atom of Arg [2]. GAA is further metabolized to creatine by guanidinoacetate *N*-methyltransferase (GAMT; EC 2.1.1.2). AGAT also catalyzes the formation of L-homoarginine (hArg) from Arg and L-lysine (Lys), with Orn being the second reaction product (reaction (b) in Scheme 1) [1]. hArg can be converted to Arg and Lys (reversed reaction (b)). Rat kidney AGAT has a relatively broad substrate specificity [3]. Other guanidino compounds such as GAA and creatine can serve as substrates (Scheme 1, reaction (c)) and/or inhibitors of AGAT activity [1]. Low circulating and low excretory concentrations of hArg are associated with worse cardiovascular outcomes and mortality [4,5,6,7,8,9,10], suggesting a particular importance of hArg in health and disease. Yet, the underlying mechanisms are still elusive and warrant further investigation.

The metabolic fate of hArg in humans and animals has been little investigated. Arginase readily hydrolyzes Arg to Orn and urea. Bovine and human arginase also catalyze the hydrolysis of hArg to Lys, yet the reaction rate is several orders of magnitude smaller than that of Arg [11,12,13]. Injection of unlabeled hArg (1880 mg hArg/kg body weight) together with radiolabeled hArg (i.e., L-[*guanidino*-^14^C]-homoarginine) into rats was found to result in the appearance of unlabeled and radiolabeled urea in the kidney and liver, suggesting arginase-dependent hydrolysis of hArg to urea [14]. Analogous to other amino acids and metabolites, hArg has been shown to be a substrate of alanine:glyoxylate aminotransferase 2 [15,16]. Pharmacological hArg was shown to be metabolized to Lys in humans and in rats [13], and to the polyamine homoagmatine in rats [17], presumably by Orn decarboxylase (ODC; EC 4.1.1.17). Free Lys is known to undergo multiple metabolism and catabolism including enzymatic decarboxylation by ODC in the heart and brain [18,19,20,21]. Furthermore, residues of Lys and Arg in proteins undergo numerous enzymatic and non-enzymatic post-translational modifications (PTM) [22]. 

Catecholamines, including the drug isoprenaline, are known to affect the ODC activity in the rat heart [23,24,25,26,27]. Isoprenaline is experimentally used to induce takotsubo cardiomyopathy (TCC) in animals [28]. In this rat model of TTC, we previously investigated the distribution and the metabolism of pharmacological high-dosed hArg in blood and main organs of the rat to metabolites of the Arg/NO pathway [17,29]. We observed high, dose-dependent concentrations of hArg in plasma, but relatively small changes in the concentration of Arg, indicating that even high-dosed hArg is not expected to severely affect Arg-involving pathways in the rat [17,29]. In rats, dietary hArg was reported to induce lysine imbalance [30]. Moreover, supplementation of creatine, a guanidino compound, in healthy humans was found to alter plasma guanidino compounds [31]. Interestingly, inter-organ amino acid interchange was observed in propionic acidemia suggesting the possible contribution of inter-organ interchange of amino acids to the physiopathology of propionic acidemia [32]. Prominent examples of inter-organ amino acid interchanges are glutathione, cysteine, and cystine [33,34]. 

In our previous study [29], we did not measure Lys and many other amino acids in blood and organ specimens of the rats administered with hArg. As Lys and hArg are mutual metabolites (Scheme 1), the focus of the present study was on the effects of hArg on Lys distribution and possible interchanges between organs. In the present study, we measured free and proteinic Lys and other free and proteinic amino acids in heart, kidney, lung, and liver specimens collected in our previous study [29] by means of validated gas chromatography-mass spectrometry (GC-MS) methods [35] and analyzed their relationships to hArg and Lys. Since isoprenaline was used in the study to induce TCC, we were also interested to obtain information about the possible effects of isoprenaline on Lys and hArg metabolism. 

## 2. Results

Table 1 summarizes the rats used in the study, the hArg doses applied, the time points of blood sampling and sacrifice, and the organs analyzed for their amino acid contents. 

### 2.1. Free Amino Acids in Tissue Homogenates

**Figure 1:**
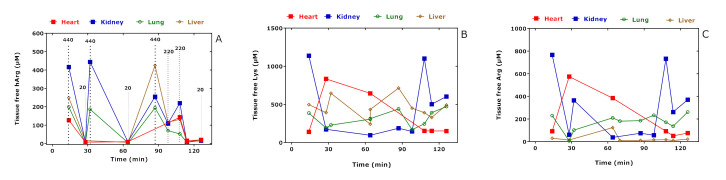
Tissue-free hArg (**A**), Lys (**B**), and Arg (**C**) concentrations in the study rats administered with the indicated hArg doses at t = 0 min and sacrificed at the indicated time points. See also Table 2.

The concentrations of the free amino acids in the organ homogenates are listed in Table S1. The sum of the concentrations of the free and proteinic amino acids in homogenates (incubated at room temperature and 110 °C for 20 h in 6 M HCl, respectively) measured in the organ homogenates are summarized in Tables S2 and S3. The concentrations of free hArg, Lys and Arg measured at various time points in the organ homogenates of the rats administered with hArg are summarized in Table 2 and illustrated in Figure 1. The concentrations (in µM) of free and total amino acids in the homogenates of the analyzed organs of the rats are listed in Table S3. 

The lowest free hArg concentrations were measured in the lung and liver of the rat R5 that did not receive hArg; similarly, low hArg concentrations are also expected for the heart and kidney of this rat (not measured). Besides free hArg (Figure 1A), the greatest changes were observed for tissue-free Lys (Figure 1B) and tissue-free Arg (Figure 1C), notably in the kidney and heart. There was no correlation between the free hArg, Lys, and Arg concentrations in the organ homogenates and the time of sacrifice of the rats when considering all data. When considering all measurements as well, only the concentration of free hArg in the tissue homogenates correlated closely with the hArg dose administered (r = 0.9165, *p* < 0.0001; *n* = 34). The hArg dose correlated strongly with the free hArg concentration in the kidney (r = 0.9354, *p* = 0.0016; *n* = 9), in the lung (r = 0.9211, *p* = 0.0008; *n* = 10) and in the liver (r = 0.9037, *p* = 0.0026; *n* = 9), but not in the heart (r = 0.7775, *p* = 0.1333; *n* = 6). The concentration–time “curves” of free Lys and free Arg in the kidneys are very similar and seem to opposite their course in the heart (Figure 1B,C) and to parallel the concentration–time “curve” of hArg in the heart (Figure 1A).

The correlation coefficients after Spearman between the tissue-free Lys concentration and the concentration of the other tissue-free amino acids are summarized in Table 3. Free Lys tissue concentration correlated with free hArg concentration positively in the kidney (r = 0.667) and liver (r = 0.683), negatively in the heart (r = −0.677), and did not correlate with free hArg in the lung. The correlation coefficients after Spearman between the hArg dose and the concentration of the other tissue-free amino acids are summarized in Table 3. Analogously, the hArg dose correlated with the tissue concentration of free Lys positively in the kidney and liver, negatively in the heart, and did not correlate with free hArg in the lung.

Linear regression analysis by considering the free Lys and free Arg concentrations measured in the hearts of all rats (except for R17, which did not receive isoprenaline) resulted in strong linear relationships between the heart concentration (y, µM) and the sacrifice time (x, min): Lys: y = 1080 − 7.8x, r^2^ = 0.9632, *p* = 0.003; Arg: y = 730 − 5.6x, r^2^ = 0.9947, *p* = 0.0017. These data allow an estimate of the cardiac half-life of 69 min for Lys and 65 min for Arg, indicating concomitant metabolism of Lys and Arg.

**Figure 2:**
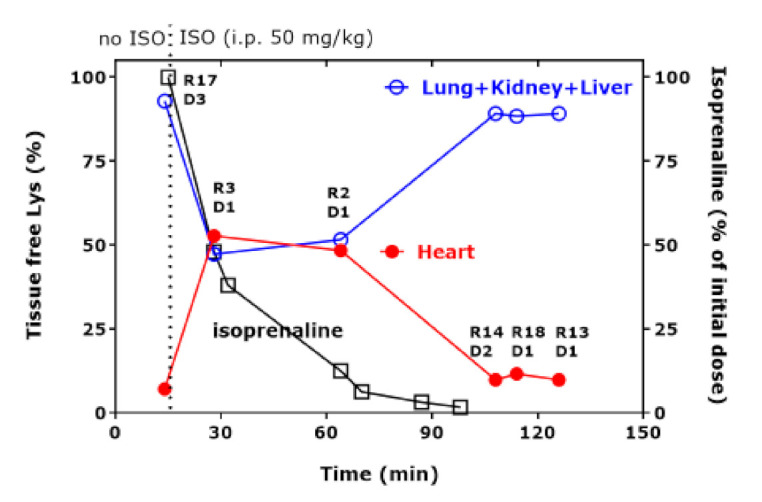
Tissue-free Lys percentage concentration in the heart and summed free Lys percentage concentration in lung, kidney, and liver of the indicated rats administered with the indicated hArg doses at t = 0 min and sacrificed at the indicated time points. The time course of percentage isoprenaline was calculated using an elimination half-life of 10 min [36]. D1, 20 mg/kg; D2, 220 mg/kg; D3, 440 mg/kg. R, rat; D, hArg dose.

Due to the potentially key role of the heart in hArg metabolism to Lys (Figure 2), we calculated the percentage changes of free Lys in the tissues of the rats in which heart data were available. R17 received the highest hArg dose but not isoprenaline because it died just before, whereas all the other rats (R2, R3, R13, R14, R18) received isoprenaline. Figure 2 shows the percentage content of free Lys in the homogenates of the heart and the summed percentage content of free Lys in the homogenates of lung, kidney, and liver of the rats. Figure 2 also shows the remaining isoprenaline percentage concentration at the indicated time points, which was calculated by considering an elimination half-life of 10 min in the rat [36]. 

Figure 2 shows the diametrical “course” of the Lys content of the heart and of the other organs together. Upon the disappearance of isoprenaline from the rat’s body at about 100 min, free Lys in the heart and the summed free Lys in the other organs did not change over time anymore. Between the time points 28 min and 64 min there seems to be an equilibrium for the percentage content of free Lys in the heart and of free Lys in the other three organs. Figure 2 suggests that free Lys is first shifted from the lung/kidneys/liver to the heart to reach an equilibrium and then in the time interval 64–108 min from the heart to the other organs, obviously without a considerable change in the blood concentration of Lys in the circulation.

### 2.2. Total Amino Acids in Tissue Homogenates

The concentrations of free hArg and of free + proteinic hArg measured in all tissues were very similar: 21.2 [8–188] µM vs. 20.1 [9.4–164] µM (*p* = 0.407, Wilcoxon test) (Tables S2 and S3). Bland–Altman analysis revealed a bias 3.9 ± 13.1 µM (95% limits of agreement, −22 to 30; four negative values). Free hArg and total hArg concentrations in all organs correlated strongly (r = 0.991, *p* < 0.0001). Linear regression analyses resulted in the regression equation y = 1.35 + 0.949x (r^2^ = 0.9919, *p* < 0.0001). These observations strongly suggest no incorporation of pharmacological hArg into tissue proteins and no guanidination of Lys residues to generate hArg residues in the rat tissue proteins under the experimental conditions. Free ADMA and total ADMA concentrations in all organs correlated moderately (r = 0.7159, *p* < 0.0001). Linear regression analyses resulted in the regression equation y = 3.7 + 4.777x (r^2^ = 0.5866, *p* < 0.0001), indicating an almost 5-fold higher proteinic ADMA concentration. A stronger correlation was observed for the sum of free and total Cit and Orn (r = 0.9309, *p* < 0.0001). Linear regression analyses resulted in the regression equation y = 48.1 + 1.184x (r^2^ = 0.9265, *p* < 0.0001), indicating quite similar free and proteinic Cit + Orn concentrations. Of the other amino acids, correlations between free and total concentrations were observed only for Met (r = 0.3773, *p* = 0.0255) and Glu + Gln (r = 0.3518, *p* = 0.0382). 

**Figure 3:**
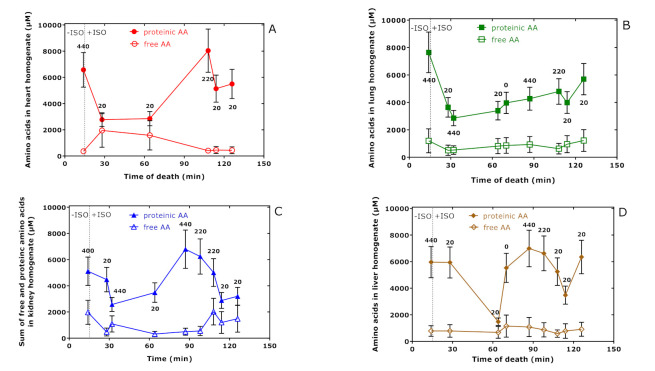
Sum of tissue-free and proteinic amino acid (AA) concentrations in heart (**A**), lung (**B**), kidney (**C**), and liver (**D**) of rats administered with the indicated hArg doses at time = 0 min and sacrificed at the indicated time points. Data are presented as the mean ± SEM. The vertical dotted lines indicate the time point of isoprenaline (ISO) injection at t = 15 min.

Figure 3 shows that the sum concentration of all proteinic amino acids measured in the tissue homogenates of the study rats behave differently, suggesting an exchange between the organs with different kinetics. The concentration of the sum of all amino acids in all organs was determined to be (mean ± SEM) 19.9 ± 2.2 mM, with Lys being the largest contributor (12.5 ± 1.9 mM) (Figure S1). 

**Figure 4:**
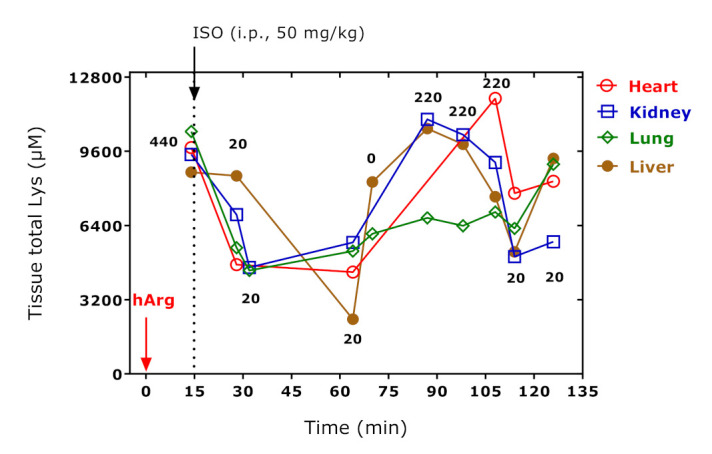
Total Lys concentrations in heart, kidney, lung, and liver of the study rats administered with the indicated hArg doses at t = 0 min and isoprenaline (ISO) at t = 15 min and sacrificed at the indicated time points. Arrows indicate the administration of hArg and isoprenaline. See Table S1.

Figure 4 shows the concentration–time “curves” for total Lys in the organs of the study rats. All curves are similar to those obtained for the sum of proteinic amino acids have a relatively broad minimum between 30 min and 64 min after hArg administration. There was no linearity between Lys concentration and time. Linearity was observed for the lung, when the 14 min value was disregarded (r^2^ = 0.69, *p* = 0.0055). The highest yet borderline correlations (after Spearman) between hArg dose and total Lys concentration were observed for the heart (r = 0.8332, *p* = 0.0667) and the liver (r = 0.6672, *p* = 0.0560) suggesting a steady increase in tissue Lys in these two organs.

### 2.3. Guanidinoacetate in Organ Homogenates

**Figure 5:**
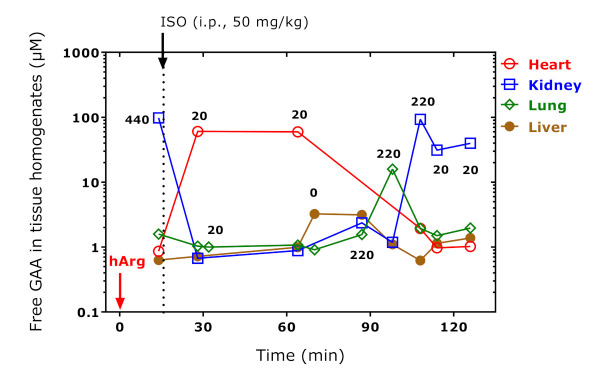
Tissue-free GAA concentration in organ homogenates of the rats. The indicated hArg doses were administered at t = 0 min and the rats were sacrificed at the indicated time points. Isoprenaline (ISO) was administered at t = 15 min. Arrows indicate the administration of hArg and isoprenaline.

The concentration of free and total GAA in the rat organs correlated (r = 0.479, *p* = 0.004), but did not differ each from other (*p* = 0.1565). Bland–Altman analysis revealed a bias of −4.5 ± 19.5 µM (95% limits of agreement, −43 to 34, seven negative values) and a systematic error: Linear regression analysis between the difference (y) and the average (x) of total and free GAA concentration yielded a straight line with the regression equation y = 6.5 − 0.97x (r^2^ = 0.7868). These observations argue for the instability of GAA under the strong acidic and thermal conditions of the proteolysis and for the lack of proteinic GAA in the analyzed organs of the rats. We, therefore, considered the concentration of free GAA. Figure 5 shows the concentration–time “curves” for free GAA in four organs of the study rats. This “kinetics” resembles that of free Lys in the same organs (Figure 2). The greatest and diametric changes were seen for GAA in the heart and kidney, whereas the GAA concentrations did not change very much in the lung and liver. GAA in the heart and lung correlated inversely (r = −0.885, *p* = 0.019, *n* = 6); the negative correlation between GAA in the heart and kidney failed statistical significance (r = −0.775, *p* = 0.070, *n* = 6). Figure 5 suggests an almost 100-fold increase in free GAA in the kidney, presumably by the reaction of Arg and hArg with Gly releasing Orn and Lys, respectively (Scheme 1). Comparable increases were observed for free GAA in the heart of the rats sacrificed at 28 and 64 min (Figure 5).

### 2.4. ADMA in Organ Homogenates—Effects on Post-Translational Arg-Dimethylation

The summed concentrations of total and free ADMA in the homogenates of all organs and time points were 16.2 ± 9.9 µM and 2.6 ± 1.6 µM, respectively. There was a moderate correlation (r = 0.766, *p* < 0.0001, *n* = 34) between total and free ADMA concentration when considering all organs and all time points. Bland–Altman analysis revealed a bias of 13.6 ± 8.7 µM (95 % limits of agreement, −3.5 to 31) with no negative values. This result suggests that the concentration of total ADMA was consistently higher than the concentration of free ADMA. In the Bland–Altman analysis, linear regression analysis between the difference (y) of total and free ADMA, i.e., the concentration of proteinic ADMA, versus the average of total and free ADMA (x), resulted in a straight line with the regression equation y = −0.82 + 1.53x, r^2^ = 0.9575. 

**Figure 6:**
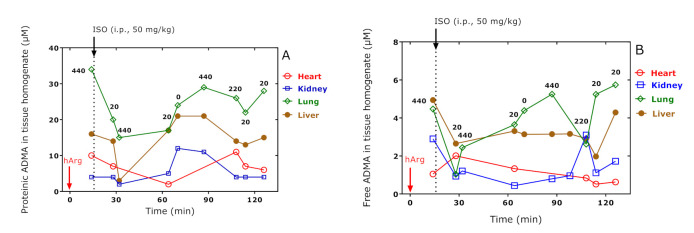
Tissue proteinic (**A**) and tissue-free (**B**) ADMA concentration in organ homogenates of the rats. The indicated hArg doses were administered at t = 0 min and the rats were sacrificed at the indicated time points. Isoprenaline (ISO) was administered at t = 15 min. Arrows indicate the administration of hArg and isoprenaline.

The concentration–time “profiles” for proteinic and free ADMA in the organs of the ras obtained in the present study are shown in Figure 6. Administration of the highest dose of hArg (i.e., 440 mg/kg) in R17 which was sacrificed at 14 min and was not treated with isoprenaline resulted in proteinic ADMA with the highest concentration being observed in the lung followed by the liver, heart, and kidney (Figure 6A). This order differed from that observed for free ADMA: liver ≈ lung, kidney, and heart (Figure 6B). It can be assumed that this distribution was independent of isoprenaline because R17 did not receive any isoprenaline. The concentration–time “course” of ADMA behaved almost diametrically only in the heart. Moreover, the free ADMA concentration in the lung and heart correlated inversely each with other (r = −0.8393, *p* = 0.037, *n* = 6). The lowest value for proteinic ADMA was observed at 64 min, whereas the highest value for free ADMA was obtained at 28 min. The cardiac half-life of free ADMA is estimated from the data of Figure 6B to be about 80 min.

### 2.5. Proline and Hydroxy-Proline in Organ Homogenates—Effects on Post-Translational Pro-Hydroxylation 

The summed concentrations of total and free Pro in the homogenates of all organs and time points were 4552 [3350–5787] µM and 249 [123–327] µM respectively. There was no correlation between total and free Pro concentration when considering all organs and time points. Bland–Altman analysis revealed a bias of 4265 ± 1387 µM (95% limits of agreement, 1547 to 6983) with no negative values. This result suggests that the concentration of total Pro was consistently higher than the concentration of free Pro. In the Bland–Altman analysis, linear regression analysis between the difference (y) of total and free Pro, i.e., the concentration of proteinic Pro, versus the average of total and free Pro (x) resulted in a straight line with the regression equation y = 32 + 1.72x, r^2^ = 0.8054.

The summed concentrations of total and free OH-Pro in the homogenates of all organs and time points were 46.5 [28–144] µM and 15.4 [10.5–19.3] µM, respectively. There was no correlation between total and free OH-Pro concentration when considering all organs and time points. Bland–Altman analysis revealed a bias of 100 ± 195 µM (95% limits of agreement, −282 to 483) with no negative values. This result suggests that the concentration of total OH-Pro was consistently higher than the concentration of free OH-Pro. In the Bland–Altman analysis, linear regression analysis between the difference (y) of total and free OH-Pro, i.e., the concentration of proteinic Pro, versus the average of total and free OH-Pro (x) resulted in a straight line with the regression equation y = −33 + 1.97x, r^2^ = 0.9833.

The summed molar ratio of total OH-Pro to total Pro in the homogenates of all organs and time points was 0.013 [0.009–0.028]. The summed molar ratio of free OH-Pro to free Pro in the homogenates of all organs and time points was 0.065 [0.040–0.093]. Bland–Altman analysis revealed a bias of −0.05 ± 0.039 µM (95% limits of agreement, −0.127 to 0.025) with all values being negative. In the Bland–Altman analysis, linear regression analysis between the difference (y) of total OH-Pro to total Pro, and of total OH-Pro to total Pro, versus the average of the ratios (x) resulted in a straight line with the regression equation y = −0.025 − 0.522x, r^2^ = 0.1526 (*p* = 0.0224). The molar ratio of total Pro to free Pro was estimated to be 20.1 [11.4–36.2]. The molar ratio of total OH-Pro to free OH-Pro was calculated to be 2.69 [1.89–10.8]. The molar ratio of the ratio of total OH-Pro to total Pro to the ratio of free OH-Pro to free Pro was determined to be 0.21 [0.14–0.40]. The hArg dose correlated solely with the total Pro concentration (r = 0.401, *p* = 0.019).

**Figure 7:**
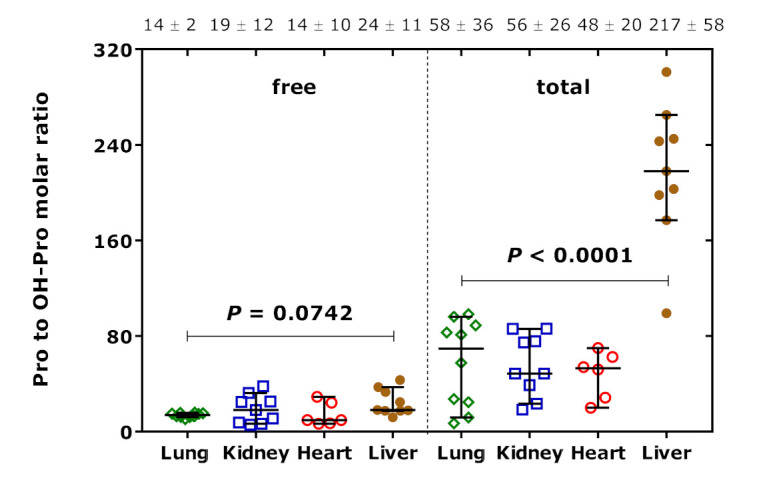
Molar ratio of Pro to OH-Pro in their free and proteinic forms in organ homogenates of the rats. Data are shown as the median with 95% confidence interval (*n* = 6–10). One-way ANOVA was performed in both cases. The numbers on the top of the Figure are the mean ± standard deviation values of the respective molar ratios.

Figure 7 shows the molar ratio of Pro to OH-Pro (Pro/OH-Pro) in their free and proteinic forms in organ homogenates of the rats. The free Pro/OH-Pro ratio did not differ between the organs. The total Pro/OH-Pro ratio did differ in the liver, and total Pro correlated closely with free Pro (r = 0.867, *p* = 0.005). None of the Pro parameters measured in the liver did correlate with the hArg dose, presumably due to the lack of an effect of pharmacological hArg on Pro metabolism. It seems that the extent of the hydroxylation of Pro residues in liver proteins is about 3 to 4 times lower than in the other rat organs.

### 2.6. Citrulline und Ornithine—Effects on Post-Translational Citrullination and Arginase Activity

**Figure 8:**
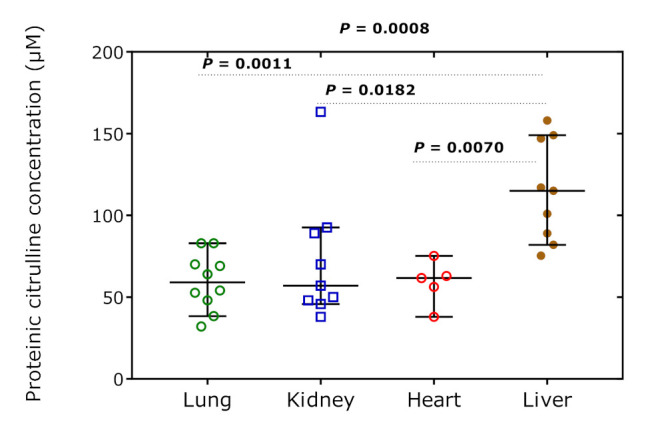
Concentration of Cit in the rat tissue organs over all time points. Data are shown as the median with 95% confidence interval (*n* = 5–10). One-way ANOVA with Tukey’s multiple comparisons test was performed.

The GC-MS method used in the present study measures the sum of Cit and Orn because Cit is converted to Orn during the derivatization procedures [35]. Thus, the utility of proteinic Cit as a measure of citrullination in proteins is limited. Nevertheless, under the assumptions that Orn, which is not a proteinogenic amino acid, and that Orn cannot be formed under the conditions of the animal experiment, the extent of citrullination can be estimated. Figure 8 shows the calculated concentration of Cit the proteins of the rat organs. The greatest difference is seen between the liver and the other organs. This observation may suggest that the extent of citrullination is about two times higher in the liver compared to the lung, kidney, and heart. The citrullination was independent of the hArg dose and of the sacrifice time in all organs (data not shown).

**Figure 9:**
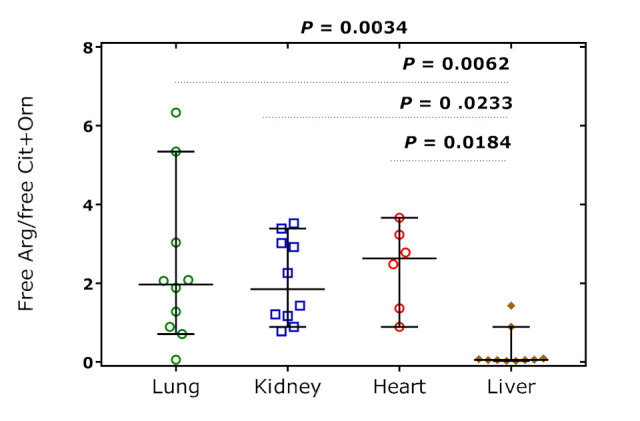
Molar ratio of free Arg to the summed concentration of free Cit and Orn, the global arginine bioavailability ratio (GABR), in the rat tissue organs over all time points. Data are shown as the median with 95% confidence interval (*n* = 6 or 10). One-way ANOVA was performed (*p* = 0.0034) with Tukey’s multiple comparisons test.

The values of the global arginine bioavailability ratio (GABR), i.e., the molar ratio of free Arg concentration to the sum concentration of free Cit and Orn, were 1.97 [0.85–3.61] in the lung, 1.85 [1.1–3.1] in the kidney, 2.63 [1.24–3.39] in the heart, and 0.055 [0.045–0.29] in the liver (Figure 9). This observation would indicate a very high arginase activity in the rat liver. GABR was independent of the hArg dose and sacrifice time (data not shown).

### 2.7. Glutamate und Glutamine

The GC-MS method used in the present study measures the sum of Glu and Gln because Gln is converted to Glu during the derivatization procedures [35]. The results for the sum of Glu and Gln are summarized in Tables S1–S3. When considering all data, there were weak positive correlations between total Glu + Gln, free Glu + Gln, and hArg (data not shown). The concentrations of total Glu + Gln, on the one side, and those of free Glu + Gln, on the other side, did not differ in the organs. This also applies to their molar ratio. Linear regression analysis between the difference of total Glu + Gln and free Glu + Gln concentration (y) and their average concentration (x) resulted in high linearity in all organs. They were as follows: y = −70 + 1.61x (r^2^ = 0.942, *p* < 0.0001) for the lung; y = −498 + 1.62x (r^2^ = 0.871, *p* = 0.0002) for the kidney; y = −3932 + 2.11x (r^2^ = 0.983, *p* = 0.0001) for the heart; and y = −1707 + 1.92x (r^2^ = 0.996, *p* < 0.0001) for the liver. We tested correlations between the hArg dose and the concentrations of total and free Glu + Gln in the rat organs. There were correlations after Spearman in the lung. The hArg dose correlated with free Glu + Gln only in the kidney (r = 0.802, *p* = 0.016). When considering all data, hArg dose correlated weakly both with free Glu + Gln (r = 0.368, *p* = 0.032) and total Glu + Glu (r = 0.420, *p* = 0.014). A correlation between free and total Glu + Gln was found only for the lung (r = 0.733, *p* = 0.020). 

### 2.8. Correlations between Free Amino Acids in Organ Homogenates

**Figure 10:**
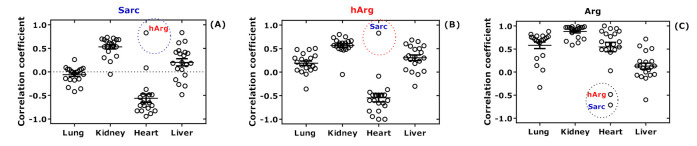
Spearman correlation coefficients for free tissue Sarc (**A**), hArg (**B**), and Arg (**C**) versus the other free tissue amino acids in lung, kidney, heart, and liver of the study rats. The complete set of the results are found in Figure S2. All amino acids are represented by a symbol. The data points for Sarc and hArg are encircled.

We performed additional statistical analyses to investigate potential relationships among the amino acids. First, separate correlations after Spearman were performed between the concentrations of the free amino acids measured in the homogenates in rat organs. Then, the correlation coefficients obtained from those analyses were plotted for each amino acid and the four organs. The relationships for Sarc, hArg, and Arg are separately illustrated in Figure 10. The results of these analyses for all amino acids are summarized in Figure S2. In the hearts of all rats, the correlation coefficients were negative for Sarc (Figure 10A) and hArg (Figure 10B) and positive for Arg (Figure 10C). In the hearts as well, there was a positive correlation between Sarc and hArg, but negative between Arg with hArg or Sarc. Solely for Met in the hearts, there was no correlation. These analyses suggest a close association of Sarc with hArg and Arg in the rat hearts.

### 2.9. Effects on the AGAT-Catalyzed Reactions and Equilibria

**Figure 11:**
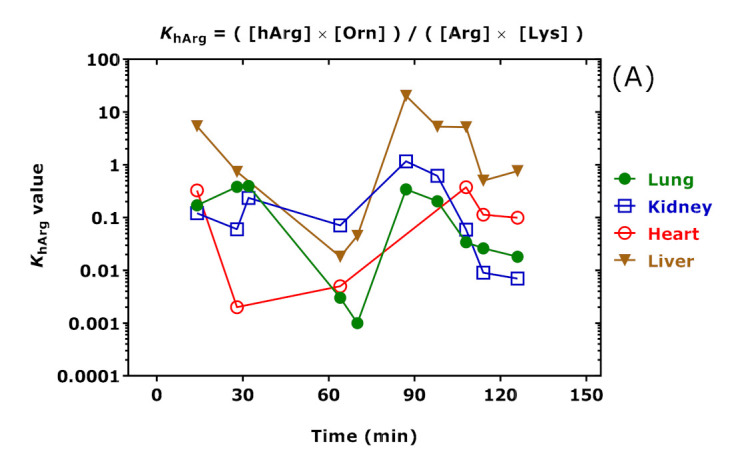

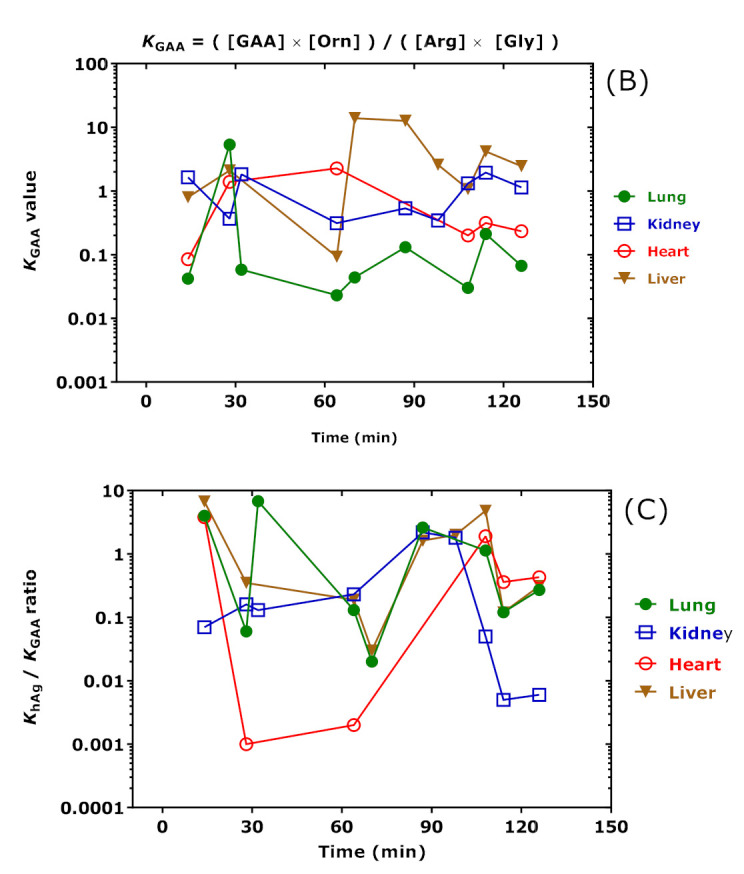
(**A**) Equilibrium constant (*K*_hArg_) values of the AGAT-catalyzed reaction Arg + Lys ↔ hArg + Orn; (**B**) equilibrium constant (*K*_GAA_) values of the AGAT-catalyzed reaction Arg + Gly ↔ GAA + Orn; and (**C**) *K*_hArg_/*K*_GAA_ values in the organs of the rats at the time of sacrifice upon i.p. administration of the indicated doses of hArg. The Figure was constructed using the data listed in Table 4. Note the logarithmic scale on the *y* axes.

AGAT catalyzes the reaction Arg + Lys ↔ hArg + Orn (see Figure 1). The equilibrium constant *K*_hArg_ of this reaction is calculated by using the concentrations (set in square brackets) of the free amino acids Arg, Lys, hArg, and Orn measured in the rat organs at the time of sacrifice: *K*_hArg_ = ([hArg] × [Orn])/([Arg] × [Lys]) are listed in Table 4 and shown in Figure 11A for clarity. AGAT also catalyzes the reaction Arg + Gly ↔ GAA + Orn (see Figure 1). The equilibrium constant *K*_GAA_ of this reaction is calculated by using the concentrations (set in square brackets) of the free amino acids Arg, Gly, GAA, and Orn measured in the rat organs at the time of sacrifice: *K*_GAA_ = ([GAA] × [Orn])/([Arg] × [Gly]) are listed in Table 4 and shown in Figure 11 for clarity. Because of methodological reasons [35], [Orn] is actually the sum of free Orn and Cit concentrations. 

The *K*_hArg_ and *K*_GAA_ values changed over time in dependence of the hArg dose. The Spearman correlation coefficients between *K*_hArg_ and hArg dose were *r* = 0.706 (*p* = 0.03) for the lung, *r* = 0.739 (*p* = 0.03) for the kidney, *r* = 0.778 (*p* = 0.13) for the heart, and *r* = 0.905 (*p* = 0.002) for the liver. The Spearman correlation coefficients between *K*_GAA_ and hArg dose were inverse and borderline: *r* = −0.845 (*p* = 0.07) for the heart. The highest *K*_hArg_ and *K*_GAA_ values and greatest changes were observed in the liver. These findings suggest that the liver may play a particular role in the catabolism of pharmacological hArg. The highest correlation after Spearman was observed between liver and lung for *K*_hArg_ (*r* = 0.683, *p* = 0.05; see Figure 11A). The highest correlation after Spearman was observed between the kidney and heart for *K*_GAA_ but failed statistical significance (*r* = −0.657, *p* = 0.18). Interestingly, the “time-courses” of *K*_hArg_ and *K*_GAA_ were opposite in the heart (Figure 11). The “time-course” of the *K*_hArg_/*K*_GAA_ ratio in the heart indicates that the greatest decreases were observed at 28 min and 64 min, presumably due to the presence of isoprenaline in this time window. The *K*_hArg_/*K*_GAA_ ratio corresponds to the product ([hArg]/[GAA]) × ([Gly]/[Lys]).

### 2.10. Putrescine and Spermidine in Organ Homogenates 

**Figure 12:**
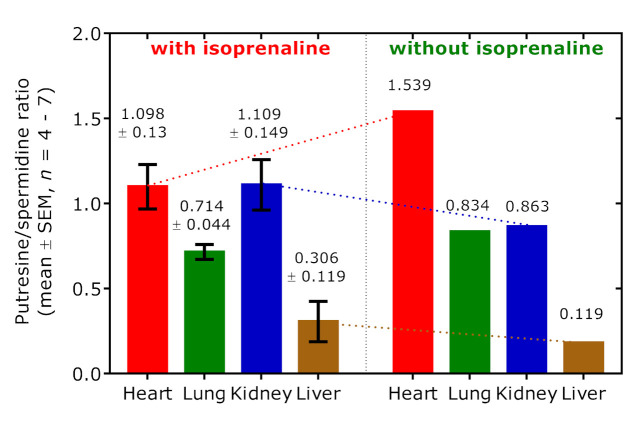
Putrescine-to-spermidine molar ratio in the organs of the rats administered with isoprenaline (**left panel**) and in a rat (R17) that did not receive isoprenaline (**right panel**). The rats received various doses of hArg. R17 received an hArg dose of 440 mg/kg. See also Table 1.

Polyamine putrescine (PUT) is the decarboxylation product of Orn and is further metabolized to spermidine (SPD). The putrescine:spermidine (PUT/SPD) molar ratio is generally considered a useful indicator of polyamine catabolism. In the rat R17, which did not receive isoprenaline but did receive the highest hArg dose, the PUT/SPD molar ratio was highest in the heart, very close in the lung and kidney, and lowest in the liver 14 min after hArg administration (Figure 12). When considering all other rats, the mean PUT/SPD molar ratio was lower in the heart (1.1 vs. 1.5; *n* = 4) and in the lung (0.71 vs. 0.83, *n* = 7), higher in the kidney (1.11 vs. 0.86, *n* = 6) and in the liver (0.31 vs. 0.12, *n* = 6) in the rats administered with isoprenaline. 

## 3. Discussion

There is evidence of significant inter-organ amino acids transport by blood cells rather than by plasma in humans [37,38]. Blood contributes substantially to the net flux of amino acids from the muscle and gut to the liver in humans, with Ala predominating by way of blood cells as well as of plasma [37]. Dietary hArg at large amounts administered for several days was found to induce imbalances in the homeostasis of various biochemically closely related (Lys, Orn, Arg, Gly) and other less closely related amino acids in plasma and in several organs of fed rats [30]. Dietary hArg was found to completely prevent the rise of Lys concentration in the brain [30]. This effect was less pronounced for Arg and Orn in the brain as well as in the liver and in the muscle of the rats. Chronic oral supplementation of large amounts of creatine to healthy humans was found to alter plasma guanidino compounds including hArg, Arg, and GAA, yet to a much lower extent [31]. An inverse relationship between plasma GAA and plasma creatinine was observed, suggesting creatine as a regulator of AGAT activity rather than of AGAT expression in humans [31]. Yet, given the supplementation of creatine at high amounts, the observed changes should be considered rather minor, presumably due to the low repressor effect of creatine on the AGAT activity. In the present study, we investigated the effects of pharmacological hArg on the amino acids’ homeostasis in the liver, kidney, heart, and lung in the rat model of isoprenaline-induced TTC. For this, we measured the concentrations of free and proteinic amino acids in the remaining specimens collected previously [29]. 

In the rat model of isoprenaline-induced TTC [28], a single i.p. injection of hArg (20, 220, 440 mg hArg per kg body weight; 440 mg hArg ≈ 2.3 mmol) resulted in the dose-dependent distribution of hArg in plasma and investigated organs [29]. The hArg plasma concentration ranged between about 2 µM and 1300 µM, and hArg disappeared from the rat plasma with a half-life in the order of 20 to 40 min [29]. Despite the high i.p. doses, hArg resulted in relatively small changes in the tissue and free plasma concentrations of its relatives, i.e., Arg, ADMA, and GAA [29]. The plasma Arg concentration ranged between 160 µM and 260 µM, suggesting Arg as a major metabolite of hArg [29]. The changes in nitrite and nitrate, the major metabolites of Arg-derived nitric oxide (NO), were marginal. In this rat model of TTC, we also observed a close correlation between tissue hArg and its decarboxylation product homoagmatine (hAgm): *r* = 0.904 (*p* = 7.3 × 10^−12^) [17]. This observation strongly suggests the ODC-catalyzed conversion of hArg to hAgm, albeit to a low extent (34.8 [4.6–270] nM; range, 0.2 to 698 nM). Correlations were also observed between hAgm and Glu + Gln (*r* = 0.489, *p* = 0.0061) or hAgm and Met (*r* = 0.416, *p* = 0.022), suggesting a role of these amino acids in the expression and/or activity of ODC and presumably of other decarboxylases. In healthy subjects, oral administration of low-dosed hArg increased plasma free Lys concentration suggesting the conversion of pharmacological hArg to Lys [13] as observed previously for dietary hArg [30]. In the rat, we found that endogenous hArg and Lys are closely interrelated, independent of rat age and diet [39]. In the present study, we investigated the effects of i.p.-administered hArg to anesthetized rats on the distribution of several free and proteinic amino acids in the main organs of the rat. The focus of our study was on Lys being a major metabolite of hArg.

With the exception of hArg, the plasma concentration of free amino acids did not change considerably in the study, presumably due to their rapid transport through the rapid circulating blood. The tissue concentration of free Lys, hArg, and Arg ranged between 96 µM and 1138 µM, 0.6 µM and 444 µM, and 7.3 µM and 767 µM, respectively. Ranges in this order of magnitude and even higher have been reported in rats administered chronically with large amounts of Lys and hArg [30]. The time-dependent opposite changes of considerable extent observed for the concentration of free hArg and Lys in the kidney and heart suggest that administered hArg is distinctly managed than Lys in the rat in these organs, and that dietary hArg and its metabolite Lys are closely interrelated. In our study, we did not analyze the rat brain for amino acids so we cannot confirm an opposite interaction of co-administered Lys and hArg in the rat brain [30]. Considerable changes in tissue amino acid concentrations were observed in our study in the time interval in which isoprenaline is present in the rats. Our results suggest that isoprenaline may be responsible for shifts in free and proteinic amino acids in the rat organs. It seems that isoprenaline contributed to the temporary increase in the Lys content in the heart until 60 min and a concomitant decrease in the Lys content in the other organs in the same period. The greatest and opposite changes in the contents of free and proteinic amino acids occurred in the heart, possibly suggesting that significant proteolysis may have occurred followed by protein synthesis. Isoprenaline, at concentrations of 10 nM to 1000 nM, was found to increase cardiac troponin I (a Lys-rich protein) via release and degradation in isolated rabbit heart [40]. These concentrations are presumably much lower than the isoprenaline concentrations in our study. Isoprenaline was also found to proteolyze troponin I in vivo in the rat [41]. In isoproterenol-induced cardiac hypertrophy in mice, the proteasome function was found to be altered after 30 min [42]. The lysyl oxidase (LOX) family catalyzes the conversion of lysine residues in proteins into highly reactive aldehydes that form cross-links in extracellular matrix proteins. LOX is considered to play a role in cardiac function and disease [43] and may be involved in the isoprenaline-induced TTC [44]. The effects of isoprenaline on proteolysis and LOX in the isoprenaline-induced TTC were not addressed in the present study and warrant further investigations. 

Lys, Arg, hArg, Gly, and GAA are closely inter-connected in the AGAT pathway (Scheme 1). Our results suggest that GAA, a non-proteinic guanidino amino acid, behaved analogous to Lys with drastic (almost 100-fold) inter-changes in the heart and kidney. This finding may be an indication for the attempt of the rat organism to support the heart with energy, as GAA may compensate the heart in case of insufficient creatine provision [45,46,47]. 

In addition to the amino acids discussed above, we observed changes in other amino acids, including Glu/Gln, Cit/Orn, Pro, and Arg that argue for multiple effects of hArg and isoprenaline on other pathways including the metabolism of free amino acids and post-translational modifications (PTM) of amino acid residues in proteins including citrullination [22]. Arg-dimethylation and citrullination are two major PTMs that play important roles in many diseases including heart failure and arthritis [22,48]. In the present study, we observed comparable citrullination levels in the heart, kidney, and lung, which were on average about two times lower than in the liver. As citrullination was independent of the hArg dose, hArg and isoprenaline are likely not to modulate citrullination in the rat. The molar ratio of free Arg to the sum of free Cit and Orn is a measure of global Arg bioavailability (GABR). In contrast to citrullination, the GABR was lowest in the liver suggesting high arginase activity in this organ. hArg is a poor substrate for arginase [13], yet this enzyme is likely to have hydrolyzed a rather minor part of administered hArg to Lys in the liver of the rats.

Isoprenaline has been shown to regulate arginase expression [49,50,51]. In rats in which isoprenaline induced cardiac hypertrophy, long-term administration of L-arginine (800 mg/kg/day) has been reported to inhibit cardiac hypertrophy by increasing moderately NO and polyamines synthesis by elevating the expression of NO synthases and ODC, respectively [52]. In a long-term experiment in rats, isoprenaline elevated ODC activity, putrescine, spermidine, and spermine levels in the rat heart, while additional treatment with α-difluoromethylornithine (DFMO), a specific inhibitor of ODC activity and polyamine synthesis, attenuated the isoprenaline-induced effects of isoprenaline [53]. When considering all rats, which were treated with hArg and isoprenaline in our study, the mean PUT/SPD molar ratio, an indicator of polyamines synthesis, was lower in the heart and lung, higher in the kidney and liver compared to the rat treated with hArg only. Yet, the effects of isoprenaline (50 mg/kg) on polyamines synthesis are difficult to be evaluated in our study, because there was only one rat (R17) that was not treated with isoprenaline but did receive the highest hArg dose of 400 mg/kg. R17 died 14 min after hArg administration, just 1 min prior to planned isoprenaline administration. In this context, it should be noted that isoprenaline was found to elevate ODC activity and polyamine synthesis in a time-dependent manner in different regions of the rat heart [54]. In the left atrium, isoprenaline (subcutaneous 20 mg/kg) increased the PUT/SPD molar ratio from about 0.4 at baseline to the highest value of about 0.6 reached at 4 h after isoprenaline, with PUT increasing much stronger than SPD [54]. With respect to NO synthesis, the changes we observed for nitrite and nitrate in plasma and tissue were very small [29] and may indicate that administration of hArg did not influence the expression and activity of NO synthases (NOS) in our study. hArg is a substrate for NOS, yet it is also a competitor to Arg and may eventually lead to decreased NO synthesis, notably at higher hArg concentrations [55], as those measured in our study.

In humans, Lys is also catabolized to Glu, 2-amino-adipic acid, and acetyl-CoA [56]. In the skeletal muscle of the hemicorpus of the rat, isoprenaline (at 1 µM) inhibited the accumulation of many amino acids, while it increased the loss of other amino acids including Glu [57]. The isoprenaline-induced changes in amino acids were found to be in part (by about 20%) due to a decrease in protein degradation, with protein synthesis being largely unaffected by isoprenaline [57]. At high doses, Lys and Arg (each 2.64 g/day) treatment for a week was found to decrease salivary cortisol in males but not in females [58], indicating another potential action of Lys and Arg. Lys and Arg administration to rats reduced the effects of cerebral ischemic insults and inhibited Glu-induced neuronal activity [59]. It is noticeable that Lys acts like a partial serotonin receptor 4 antagonist and inhibits serotonin-mediated intestinal pathologies and anxiety in rats [60]. Whether the above-mentioned effects occurred in our study, is unknown as we did not analyze other organs of the rats besides the liver, heart, lung, and kidney. The high correlation coefficients between free Lys and Glu + Gln in the lung, kidney, and heart may suggest that Lys and its metabolite Glu/Gln [56] play a role in these organs and are affected by isoprenaline. It is noticeable that isoprenaline is assumed to have changed the rat urine metabolome [61]. The results of the present study obtained from analyses of plasma and tissue samples of isoprenaline-induced TTC in rats are supportive of this assumption including the changes seen in Sarc concentrations.

In healthy young volunteers, oral administration of hArg (125 mg/day) resulted in increases in hArg and Lys plasma concentrations without changes in pulse wave velocity (PWV), augmentation index (AIx), flow-mediated vasodilatation (FMD), corticospinal excitability, and cortical excitability [62]. In this study, plasma Lys concentration increased by about 8% compared to placebo supplementation [13]. The same dose also did not reveal any appreciable effects on cognition in healthy humans [63]. Oral administration of hArg to C57BL6 mice resulted in increased hArg plasma concentrations compared with vehicle-treated C57BL6 mice (0.15 ± 0.01 versus 0.20 ± 0.02 µM). hArg treatment decreased infarct sizes and improved neurological scores in a dose-dependent manner [64]. These observations may suggest that much higher hArg doses may be required to achieve pharmacological effects in humans than in murine models of myocardial infarction.

Potential limitations of our study are the relatively small number of rats, the lack of a control group, the lack of cardiac parameters, and the lack of tissue material from the gut, muscle, and brain that hampered a more comprehensive investigation and elucidation of underlying mechanisms. Nevertheless, our pilot study provides sufficient information for performing a more sophisticated study on the metabolism of hArg in the rat, on the effects of hArg and isoprenaline in the heart and other rat organs by measuring various biochemical and pharmacodynamic parameters. 

## 4. Materials and Methods

The distribution and the metabolism of pharmacological hArg to Arg and its metabolites from the Arg/NO pathway in a rat model of isoprenaline-induced cardiomyopathy [28] have been described in detail elsewhere [29]. Sprague-Dawley rats (10 weeks old, 250 g body weight on average) received humane care and the study protocol complied with the institutional guidelines of the Sahlgrenska University Hospital (Gothenburg, Sweden). Saline (as a control; dose D of 0 mg hArg/kg; D0) and hArg hydrochloride solutions in saline of various doses were injected intra-peritoneally (i.p.) into the anesthetized rats. Fifteen minutes after injection of saline (D0) or hArg solution in saline (D1, 20 mg/kg; D2, 220 mg/kg; D3, 440 mg/kg), isoprenaline was injected i.p. at the fixed dose of 50 mg/kg. Rats were sacrificed at varying times after a bolus dose of pentobarbital (50 mg/kg) [28]. Heart, lung, liver, and kidneys were obtained from the sacrificed rats (Table 1). Rat #17 (R17) did not receive isoprenaline because it died 14 min after hArg administration, i.e., just prior to the planned isoprenaline administration. Organs were washed with ice-cold physiological saline and stored immediately at −80 °C. After their transport on dry ice to the Hannover Medical School (Hannover, Germany), the organs were stored at −20 °C until further processing. Homogenization of weighed ice-cooled tissue (about 100 mg of each organ) was performed in 1 mL aliquots of ice-cooled phosphate-buffered saline, pH 7.2, as described previously [29]. The concentration of free and proteinic amino acids was determined by GC-MS in 10 µL aliquots of tissue homogenates [29] using trideuteromethyl esters of amino acids that served as internal standards as described elsewhere [35]. Free amino acids were measured in 10 µL aliquots of the homogenates after their incubation with 6 M HCl for 20 h at room temperature to avoid proteolysis. Total amino acids, i.e., free and proteinic amino acids, were measured after classical proteolysis in homogenates with 6 M HCl for 20 h at 110 °C. The concentration of proteinic amino acids was calculated by difference. The tissue-free polyamines putrescine and spermidine were analyzed by GC-MS as described previously after selective extraction and derivatization with pentafluoropropionic anhydride [17]. GC-MS analyses were carried out on an apparatus model ISQ from ThermoFisher (Dreieich, Germany). 

Data are reported as the mean ± standard deviation (SD) for normally distributed data and as the median with interquartile range for skewed data. Correlations between groups were performed after Spearman. Statistical analysis and preparation of graphs were performed with GraphPad Prism (version 7 for Windows, La Jolla, CA, USA).

## 5. Conclusions

Our study shows remarkable changes in free and proteinic amino acids in the rat organs. The equilibria of the AGAT-catalyzed reactions are dependent on hArg dose and time. Considerable antidromic effects were observed in the heart for the two AGAT-catalyzed reactions notably in the time window of 15–90 min, in which i.p.-administered isoprenaline is present in the rat organism. hArg and Sarc are closely and positively correlated in the heart.

## Figures and Tables

**Table 1:** Summary of the rats used in the study, the hArg doses applied, the time points of blood sampling and sacrifice, and the organs analyzed for amino acids.

Rat (R) Dose (D) ^a^	hArg Dose (mg/kg)	Time of Blood Sampling (min)	Time of Sacrifice (min)	Rat Organs Analyzed for Amino Acids
R5, D0	0	0, 15, 30, 60	70	Lung, kidney, liver, heart ^b^ (no Lys)
R2, D1	20	0, 15, 30, 60	64	Lung, kidney, liver, heart
R3, D1	20	0, 15	28	Lung, kidney, liver, heart
R13, D1	20	No sampling	126	Lung, kidney, liver, heart
R18, D1	20	0, 90	114	Lung, kidney, liver, heart
R14, D2	220	90	108	Lung, kidney, liver, heart
R19, D2	220	0, 100	98	Lung, kidney, liver, heart b (no Lys)
R1, D3	440	0, 15, 30	32	Lung, kidney, liver, heart b (no Lys)
R4, D3	440	0, 15, 30, 60	87	Lung, kidney, liver, heart b (no Lys)
R17, D3	440	0	14 ^c^	Lung, kidney, liver, heart

^a^ D0, 0 mg/kg; D1, 20 mg/kg; D2, 220 mg/kg; D3, 440 mg/kg; ^b^ the heart of rat #5 (R5) was used for histological analyses only; no data are available for heart amino acids; ^c^ rat #17 (R17) received hArg and died just before isoprenaline administration.

**Table 2:** Time of sacrifice, hArg dose, isoprenaline (ISO) administration and concentrations of free homoarginine (hArg), free lysine (Lys), and free arginine (Arg) measured in the tissue homogenates of the analyzed organs of the rats at the indicated time points. See also Table 1.

Rat No. Organ	Time of Sacrifice (min)	hArg Dose (mg/kg)	ISO Use	hArg (µM)	Lys (µM)	Arg (µM)
R17 Heart	14	440	no	127	140	92
R17 Lung	14	440	no	199	386	230
R17 Kidney	14	440	no	416	1138	767
R17 Liver	14	440	no	249	497	29.8
R3 Heart	28	20	yes	7.6	834	575
R3 Lung	28	20	yes	3.8	191	7.34
R3 Kidney	28	20	yes	8.1	173	60.7
R3 Liver	28	20	yes	15.5	392	14.3
R1 Lung	32	440	yes	186	229	102
R1 Kidney	32	440	yes	444	646	365
R2 Heart	64	20	yes	9.7	644	384
R2 Lung	64	20	yes	5.1	306	210
R2 Kidney	64	20	yes	9.7	96	37.4
R2 Liver	64	20	yes	6.3	241	124
R5 Lung	70	0	yes	0.64	313	181
R5 Liver	70	0	yes	0.62	433	9.6
R4 Lung	87	440	yes	194	441	185
R4 Kidney	87	440	yes	255	187	73.8
R4 Liver	87	440	yes	425	714	10.3
R19 Lung	98	220	yes	70	169	233
R19 Kidney	98	220	yes	109	145	56.3
R19 Liver	98	220	yes	117	451	15
R14 Heart	108	220	yes	143	154	91.2
R14 Lung	108	220	yes	52	245	171
R14 Kidney	108	220	yes	220	1101	733
R14 Liver	108	220	yes	132	391	15.9
R18 Heart	114	20	yes	15.4	153	51.3
R18 Lung	114	20	yes	7.2	391	136
R18 Kidney	114	20	yes	9.9	503	262
R18 Liver	114	20	yes	7.65	328	10.1
R13 Heart	126	20	yes	20.3	151	75.4
R13 Lung	126	20	yes	15.9	471	261
R13 Kidney	126	20	yes	15.1	602	370
R13 Liver	126	20	yes	22.1	492	21.2

**Table 3:** Spearman coefficients of correlation between the tissue concentrations (µM) of (A) free Lys and (B) free hArg with the indicated free amino acids in lung, kidney, heart, and liver of the rats.

(A) Free Lys Versus Free Amino Acids and hArg Dose					(B) Free hArg Versus Free Amino Acids and hArg Dose				
Amino acid	Lung	Kidney	Heart	Liver	Amino acid	Lung	Kidney	Heart	Liver
Ala	0.967	0.933	0.714	0.933	Ala	0.233	0.617	−0.829	0.650
Thr	0.933	0.983	0.829	0.917	Thr	−0.033	0.567	−0.714	0.600
Gly	0.683	0.95	0.829	0.444	Gly	0.333	0.617	−0.486	0.561
Val	0.967	0.983	0.829	0.733	Val	0.133	0.567	−0.486	0.417
Ser	0.95	0.967	0.657	0.483	Ser	0.050	0.650	−1.000	0.300
Sarc	0.017	0.617	−0.486	0.067	Sarc	−0.283	−0.050	0.829	0.050
Leu + Ile	0.95	0.983	0.543	0.5	Leu + Ile	0.000	0.567	−0.657	0.350
GAA	0.55	0.95	0.943	0.3	GAA	0.533	0.750	−0.600	−0.300
Asp + Asn	0.867	0.983	0.829	0.683	Asp + Asn	0.533	0.733	−0.486	0.267
OH-Pro	0.9	0.795	0.943	0.267	OH-Pro	0.500	0.469	−0.829	−0.050
Pro	0.983	0.933	0.657	0.883	Pro	0.217	0.617	−1.000	0.500
Met	0.983	0.717	0.429	0.45	Met	0.333	0.667	−0.086	0.017
Glu + Gln	0.967	0.683	0.886	0.55	Glu + Gln	0.417	0.800	−0.371	0.550
Orn + Cit	0.617	0.967	0.829	0.933	Orn + Cit	−0.067	0.550	−0.943	0.483
Phe	0.95	0.967	0.714	0.3	Phe	0.050	0.483	−0.600	0.183
Tyr	0.817	0.933	0.486	0.368	Tyr	0.067	0.517	−0.600	0.326
Arg	0.717	0.983	0.6	−0.05	Lys	0.283	0.667	−0.657	0.683
hArg	0.283	0.667	−0.657	0.683	Arg	0.300	0.583	−0.486	0.233
Trp	−0.25	0.767	0.543	−0.483	Trp	−0.217	0.500	−0.429	0.000
ADMA	1	0.9	0.6	0.5	ADMA	0.283	0.483	−0.486	0.300
hArg dose	−0.009	0.49	−0.507	0.562	hArg Dose	0.940	0.935	0.778	0.949

Abbreviations: Sarc, sarcosine; OH-Pro, hydroxy-proline; ADMA, asymmetric dimethylarginine.

**Table 4:** Equilibrium constant (*K*_hArg_) values of the AGAT-catalyzed reaction Arg + Lys ↔ hArg + Orn, equilibrium constant (*K*_GAA_) values of the AGAT-catalyzed reaction Arg + Gly ↔ GAA + Orn, and their *K*_hArg_/*K*_hArg_ ratio in the organs of the rats at the time of sacrifice upon i.p. administration of the indicated doses of hArg. n.a., not applicable.

Time (min)	hArg Dose (mg/kg)	*K_hArg_*; *K*_GAA_ and *K_hArg_*/*K*_GAA_ ratio			
		**Lung**	**Kidney**	**Heart**	**Liver**
14	440	0.17; 0.04/4.0	0.12; 1.64/0.07	0.33; 0.09/3.8	5.38; 0.80/6.7
28	20	0.31; 5.34/0.06	0.06; 0.37/0.16	0.002; 1.39/0.001	0.74; 2.11/0.35
32	440	0.40; 0.06/6.8	0.24; 1.83/0.13	n.a.	n.a.
64	20	0.003; 0.023/0.13	0.07; 0.31/0.23	0.005; 2.26/0.002	0.02; 0.09/0.19
70	0	0.001; 0.044/0.02	n.a.	n.a.	0.05; 13.9/0.03
87	440	0.34; 0.13/2.61	1.16; 0.53/2.18	n.a.	20.3; 12.6/1.61
98	220	0.20	0.62; 0.35/1.8	n.a.	5.26; 2.59/2.0
108	220	0.03; 0.03/1.13	0.06; 1.32/0.05	0.38; 0.20/1.9	5.16; 1.07/4.8
114	20	0.03; 0.21/0.12	0.009; 1.94/0.005	0.11; 0.31/0.36	0.50; 4.20/0.12
126	20	0.02; 0.07/0.27	0.007; 1.14/0.006	0.10; 0.23/0.43	0.76; 2.45/0.31

## Data Availability

The study did not report any data.

## Supplementary Materials

The following are available online at https://www.mdpi.com/article/10.3390/ijms23094734/s1.
