# Supplementary material for: Pilot Study on Acute Effects of Pharmacological Intraperitoneal L-Homoarginine on Homeostasis of Lysine and Other Amino Acids in a Rat Model of Isoprenaline-Induced Takotsubo Cardiomyopathy
Source: Int J Mol Sci. 2022 Apr 25;23(9):4734. doi: 10.3390/ijms23094734 (PMC9103764; doi:10.3390/ijms23094734)
Supplement: Supplementary file 1 [file ijms-23-04734-s001.zip › Supplement_FINAL.pdf]

Supplement

Pilot study on acute effects of pharmacological intraperitoneal L-homoarginine on homeostasis of lysine and other amino acids in a rat model of isoprenaline-induced takotsubo cardiomyopathy

Dimitrios Tsikas [1,*] and Björn Redfors [2]

[1] Institute of Toxicology, Core Unit Proteomics, Hannover Medical School, Hannover 30623, Germany

[2] Department of Cardiology, Sahlgrenska University Hospital, Gothenburg, Sweden

Table S1. Concentrations (in µM) of free amino acids in the homogenates of the analyzed organs of the rats. See Table 1.

Rat/Organ	Ala	Thr	Gly	Val	Ser	Sarc	Leu+Ile	GAA	Asp+Asn	OH-Pro	Pro	Met	Glu+Gln	Orn+Cit	Phe	Tyr	**Lys**	**Arg**	**hArg**	Trp	ADMA
**R1 Lung**	707	191	835	97.2	473	13.5	187	1.00	247	10.0	128	98.2	941	49.6	72.1	71.5	**229**	**102**	**186**	167	2.44
**R1 Kidney**	1232	824	834	710	913	10.2	863	44.6	871	15.8	509	145	1496	125	243	237	**646**	**365**	**444**	188	1.21
**R2 Heart**	1159	838	817	701	955	10.2	944	59.6	867	19.2	559	146	1521	119	254	264	**644**	**384**	**9.67**	145	1.33
**R2 Lung**	743	345	873	219	594	11.1	330	1.07	363	16.6	211	119	1125	39.3	132.2	98.5	**306**	**210**	**5.13**	165	3.65
**R2 Kidney**	481	63	197	43.5	160	6.38	96.9	0.88	215	9.79	54.9	88.4	815	26.2	41.6	43.8	**96**	**37.4**	**9.68**	145	0.44
**R2 Liver**	737	276	745	161	499	11.8	278	0.99	347	14.3	171	121	1103	86.7	111	121	**241**	**124**	**6.32**	160	3.31
**R3 Heart**	1605	1171	1190	957	1201	9.14	1439	60.5	1409	34.2	829	249	2404	157	407	329	**834**	**575**	**7.55**	141	2.01
**R3 Lung**	533	216	297	123	312	16.9	199	1.03	157	5.24	82.8	69.9	442	113	64.0	79.8	**191**	**7.34**	**3.82**	142	1.03
**R3 Kidney**	594	197	235	111	339	15.1	266	0.67	322	7.85	85.5	101	897	77.7	128	127	**173**	**60.7**	**8.12**	113	0.94
**R3 Liver**	893	535	638	336	814	30.8	543	0.72	393	6.53	243	147	1270	267	169	139	**392**	**14.3**	**15.5**	56.0	2.65
**R4 Lung**	1005	517	928	253	707	10.4	401	1.56	523	19.4	285	188	1696	144	184	197	**441**	**185**	**194**	162	5.25
**R4 Kidney**	749	196	377	94	394	9.82	198	2.36	339	17.0	110	183	1723	62.9	82.4	72.2	**187**	**73.8**	**255**	101	0.80
**R4 Liver**	1611	566	848	292	682	17.1	425	3.13	392	15.3	266	123	1110	351	160	140	**714**	**10.3**	**425**	103	3.15
**R5 Lung**	851	362	990	229	622	9.94	365	0.91	389	15.0	225	136	1152	87	150	137	**313**	**181**	**0.64**	146	4.39
**R5 Liver**	1436	522	744	291	701	29.7	453	3.25	428	14.2	256	127	1085	305	161	158	**433**	**9.6**	**0.62**	85.1	3.14
**R13 Heart**	674	188	321	93	351	12.6	208	1.02	223	10.7	102	150	1298	55.3	83.0	68.5	**151**	**75.4**	**20.3**	107	0.63
**R13 Lung**	1113	645	1548	336	858	18.1	518	1.96	588	23.3	358	222	2049	139	208	173	**471**	**261**	**15.9**	134	5.74
**R13 Kidney**	1333	821	993	651	937	12.1	812	39.7	856	22.8	574	168	1403	105	250	173	**602**	**370**	**15.1**	153	1.72
**R13 Liver**	1631	680	952	353	906	20.1	601	1.38	455	12.4	306	141	1388	358	226	149	**492**	**21.2**	**22.1**	109	4.29
**R14 Heart**	683	159	389	316	306	21.7	181	1.93	389	10.8	73.1	196	1640	36.8	75.8	63.0	**154**	**91.2**	**143**	115	0.84
**R14 Lung**	597	280	1020	140	512	14.0	244	1.94	440	15.3	159	132	1132	27.0	91.7	72.6	**245**	**171**	**52.2**	121	2.62
**R14 Kidney**	2900	1730	2074	1368	1564	21.0	2011	92.7	2119	76.4	1375	360	3445	216	567	568	**1101**	**733**	**220**	168	3.10
**R14 Liver**	954	503	883	287	740	33.5	473	0.62	388	4.59	198	106	1205	243	171	159	**391**	**15.9**	**132**	142	2.91

**R17 Heart**	740	140	363	86	334	14.2	191	0.86	324	9.02	85.3	148	1234	33.1	72.7	86.3	**140**	**92.0**	**127**	131	1.05
**R17 Lung**	980	315	1242	243	579	5.60	346	1.58	465	20.8	255	146	1654	75.9	125	116	**386**	**230**	**199**	21.9	4.47
**R17 Kidney**	2910	1759	1975	1529	1615	14.2	2167	97.8	2194	37.9	1433	348	3576	254	619	639	**1138**	**767**	**416**	172	2.90
**R17 Liver**	1659	619	848	348	872	18.4	618	0.63	480	9.13	303	126	1398	320	139	163	**497**	**29.8**	**249**	89.3	4.94
**R18 Heart**	865	156	347	71	364	19.9	161	0.97	315	19.1	124	151	1236	57.7	67.5	55.1	**153**	**51.3**	**15.4**	80.7	0.52
**R18 Lung**	1099	483	1006	278	656	21.1	430	1.50	436	18.4	288	172	1384	192	181	91	**391**	**136**	**7.21**	122	5.24
**R18 Kidney**	1166	668	711	424	849	17.7	684	31.2	650	18.4	457	134	1199	116	196	219	**503**	**262**	**9.93**	127	1.11
**R18 Liver**	847	389	581	195	538	8.04	323	1.14	273	10.9	190	100	832	216	117	139	**328**	**10.1**	**7.65**	113	1.97
**R19 Lung**	1066	484	1455	312	809	10.76	454	2.0	521	26.1	317	182	2021	115	169	234	**169**	**233**	**70**	317	4.62
**R19 Kidney**	790	116	282	89	343	6.86	150	1.18	334	15.8	118	146	1551	46.4	67.6	89.3	**145**	**56.3**	**109**	69.7	0.96
**R19 Liver**	1472	541	862	276	689	19.1	431	1.10	351	15.4	272	163	1214	304	144	101	**451**	**15.0**	**117**	135	3.16
**Median**	954	483	835	276	656	14.2	401	1.44	392	15.3	243	146	1270	116	144	137	**391**	**92**	**20.3**	131	2.62

Table S2. Concentrations (in μM) of total amino acids (free+proteinic) in the homogenates of the analyzed organs of the rats. See Table 1.

**Rat/Organ**	Ala	Thr	Gly	Val	Ser	Sarc	Leu+Ile	GAA	Asp+Asn	OH-Pro	Pro	Met	Glu+Gln	Orn+Cit	Phe	Tyr	**Lys**	**Arg**	**hArg**	Trp	ADMA
**R1 Lung**	5574	3509	4888	4390	4093	1.67	7857	3.56	6073	29.24	2811	909	7897	88	2334	1115	**4459**	**2391**	**165.41**	32.9	17.91
**R1 Kidney**	5788	3870	5037	4934	4711	1.83	8921	22.53	6473	38.93	2909	1024	8145	175	2632	1422	**4590**	**2771**	**431.73**	38.1	2.96
**R2 Heart**	5401	3858	4941	4802	4262	1.93	8608	29.52	6233	43.35	3030	812	7882	157	2549	1388	**4386**	**2797**	**9.48**	19.4	3.24
**R2 Lung**	6494	4291	5982	5284	4955	2.28	9575	3.99	7122	41.36	3353	1015	9459	92	2762	1318	**5299**	**2989**	**6.18**	32.6	20.79
**R2 Kidney**	6720	4692	5744	5285	4665	1.99	10679	3.03	7743	179.08	3274	1228	11443	72	2916	1716	**5665**	**3645**	**10.19**	27.5	5.33
**R2 Liver**	3069	2052	2933	2473	2628	2.03	4447	2.67	3070	16.67	1649	565	4124	162	1370	793	**2358**	**1379**	**4.59**	39.2	6.80
**R3 Heart**	5665	3781	4585	4623	4009	1.97	8788	3.99	6136	45.14	2823	978	8813	141	2514	1224	**4709**	**2647**	**9.55**	24.8	8.51
**R3 Lung**	6705	4372	6061	5325	5088	1.81	9629	4.41	7670	59.80	3441	1056	9512	145	2859	1364	**5439**	**3104**	**7.68**	19.5	21.52
**R3 Kidney**	8338	6111	7833	7463	6554	1.88	13732	30.47	10446	94.53	4581	1380	13162	241	3975	2390	**6862**	**4531**	**9.08**	32.5	5.11
**R3 Liver**	10499	7372	9505	9247	7786	2.10	16549	4.88	12815	21.62	5720	1657	15992	414	4983	2873	**8534**	**5368**	**18.50**	17.7	16.40
**R4 Lung**	8231	5296	7620	6283	6011	2.13	11780	4.48	9153	152.69	4159	1328	12200	208	3311	1804	**6729**	**4052**	**167.92**	34.2	34.07
**R4 Kidney**	13195	8976	10851	10634	8747	2.10	20741	4.61	15765	264.12	6162	2193	22508	152	5680	3396	**10970**	**6998**	**235.27**	33.5	13.18
**R4 Liver**	12756	8528	11504	10829	8755	2.60	19575	5.36	14973	26.34	6390	1810	18185	440	5797	3124	**10571**	**6307**	**417.28**	19.2	24.33
**R5 Lung**	7653	4929	7248	5905	5514	1.73	10976	3.38	8155	159.57	3937	1290	11064	170	3041	1539	**6042**	**3664**	**0.99**	18.5	28.29
**R5 Liver**	10445	6837	9143	8619	7142	2.55	16016	3.83	11657	26.41	5218	1498	14484	420	4579	2638	**8273**	**4948**	**0.75**	38.2	20.38
**R13 Heart**	10068	6725	8249	7924	6530	2.16	15431	4.09	11228	83.89	4523	1696	15970	117	4221	2279	**8305**	**5069**	**21.54**	19.4	6.77
**R13 Lung**	10938	7169	9908	8795	7715	1.92	16152	5.02	12867	54.97	5396	1665	15758	222	4673	2232	**9036**	**4784**	**16.90**	18.5	33.44
**R13 Kidney**	7139	4761	6156	6029	5129	1.68	10925	21.52	8369	47.33	3578	1176	9552	153	3108	1771	**5694**	**3395**	**15.57**	14.9	5.65
**R13 Liver**	11883	7964	10625	10039	8284	2.12	18124	4.60	13192	24.73	6069	1777	17203	475	5417	2908	**9288**	**5679**	**18.62**	20.0	19.02
**R14 Heart**	13752	9673	11829	11222	9129	2.21	22114	4.39	17188	121.67	6323	2447	24443	112	5989	3489	**11873**	**7500**	**160.36**	18.1	11.57
**R14 Lung**	8946	5688	9303	7116	6401	1.60	12848	4.96	10068	398.40	4707	1337	12486	81	3630	1831	**6977**	**4349**	**54.02**	20.2	28.21
**R14 Kidney**	11314	7826	10271	9632	8596	1.91	17448	35.64	13999	122.46	5950	1778	16807	286	4927	2932	**9113**	**5996**	**194.81**	18.2	6.93
**R14 Liver**	9239	6531	8850	8272	7005	2.04	14908	4.16	11073	16.65	5006	1583	14200	344	4376	2289	**7634**	**4677**	**126.64**	18.0	16.59

**R17 Heart**	11909	7849	9876	9429	7897	2.01	18367	3.95	13403	268.89	5361	2075	18821	96	5012	2859	**9755**	**5925**	**121.30**	22.9	10.92
**R17 Lung**	14271	8635	16720	11222	9741	1.82	20255	5.95	15399	1109.77	7668	2050	19071	145	5642	2860	**10451**	**6805**	**163.45**	19.1	38.02
**R17 Kidney**	11852	7946	9963	9983	8914	1.75	18117	36.27	14468	71.64	6168	1804	16668	292	5192	2940	**9457**	**5866**	**379.78**	16.5	6.53
**R17 Liver**	11149	7320	9959	9093	7766	2.24	17026	4.13	12600	26.34	5744	1824	16221	478	4963	2752	**8696**	**5531**	**264.14**	17.9	20.65
**R18 Heart**	9324	6375	7674	7095	6173	2.54	14318	4.38	10212	149.86	4258	1705	15381	114	3849	2367	**7786**	**4986**	**15.02**	28.7	7.64
**R18 Lung**	7596	5066	6785	5914	5687	2.02	11226	3.68	8856	44.25	3936	1253	11339	262	3222	1670	**6272**	**3623**	**6.88**	22.5	27.06
**R18 Kidney**	6329	4300	5553	5138	4705	2.15	9719	19.15	6955	37.97	3260	988	8824	173	2826	1575	**5053**	**3136**	**10.67**	22.4	5.40
**R18 Liver**	6631	4369	6291	5344	4696	2.23	9655	2.89	7006	18.84	3342	1086	9322	298	2917	1578	**5262**	**3115**	**7.43**	35.4	14.52
**R19 Lung**	7788	5001	7491	6072	6017	2.03	11494	3.30	8976	45.75	3799	1279	11658	163	3282	1525	**6385**	**3617**	**56.16**	38.4	26.34
**R19 Kidney**	12180	8255	10003	9524	8102	2.17	19308	5.05	13936	142.69	5550	2087	20733	139	5184	3112	**10310**	**6594**	**126.09**	36.1	11.62
**R19 Liver**	12908	7578	11540	9839	8830	2.37	18071	5.09	13994	29.15	5916	1765	17754	453	5341	2816	**9897**	**5863**	**130.67**	59.5	24.42
**Median**	9093	6243	8041	7290	6466	2.03	14025	4.45	10329	46.5	4552	1439	13681	167	3912	2256	**7306**	**4604**	**20.1**	22.5	15.5

Table S3. Mean concentrations (in µM) of free and total amino acids in the homogenates of the analyzed organs of the rats. See Table 1.

	**Ala**	**Thr**	**Gly**	**Val**	**Ser**	**Sarc**	**Leu+Ile**	**GAA**	**Asp+Asn**	**OH-Pro**	**Pro**	**Met**	**Glu+Gln**	**Orn+Cit**	**Phe**	**Tyr**	**Lys**	**Arg**	**hArg**	**Trp**	**ADMA**
**FREE**																					
**Lung**	869	384	1019	223	612	13.1	347	1.5	413	17.0	231	147	1360	98	138	127	314	172	73	150	**3.9**
**Kidney**	1351	708	853	558	790	12.6	805	**34.6**	878	**24.6**	524	186	1789	114	244	241	510	303	**165**	137	1.5
**Heart**	954	442	571	371	585	14.6	521	**20.8**	588	17.2	295	173	1556	76	160	144	346	212	54	120	1.1
**Liver**	1249	515	789	282	716	21.0	461	1.4	390	11.4	245	128	1178	**272**	155	141	438	28	**108**	110	**3.3**
**TOTAL**																					
	**Ala**	**Thr**	**Gly**	**Val**	**Ser**	**Sarc**	**Leu+Ile**	**GAA**	**Asp+Asn**	**OH-Pro**	**Pro**	**Met**	**Glu+Gln**	**Orn+Cit**	**Phe**	**Tyr**	**Lys**	**Arg**	**hArg**	**Trp**	**ADMA**
**Lung**	8420	5396	8201	6631	6122	1.9	12179	4.3	9434	**210**	4321	1318	12044	158	3476	1726	6709	3938	65	26	**27.6**
**Kidney**	9206	6304	7935	7625	6680	1.9	14399	**19.8**	10906	111	4604	1518	14205	187	4049	2362	7524	4770	**157**	27	7.0
**Heart**	9353	6377	7859	7516	6333	2.1	14604	8.4	10733	119	4386	1619	15218	123	4022	2268	7802	4821	56	22	8.1
**Liver**	9842	6506	8928	8195	6988	2.3	14930	4.2	11153	23	5006	1507	14165	**387**	4416	2419	7835	4763	**110**	29	**18.1**

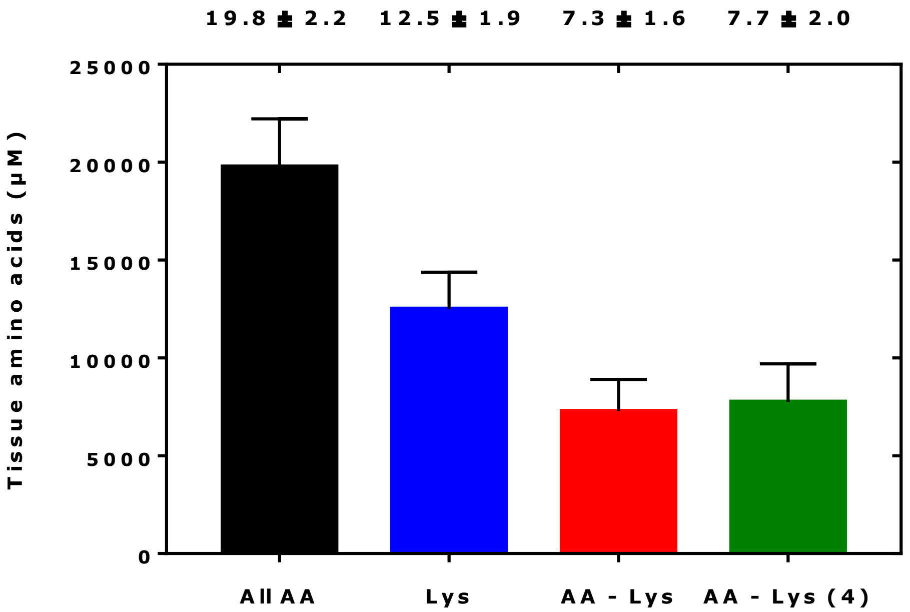

Figure S1. Concentration of the sum of all amino acids (All AA), of lysine (Lys), of all amino acids except for Lys (AA-Lys) in all organs available, and of all amino acids except for Lys in the four organs of six rats (AA-Lys (4)). See also Table S1.

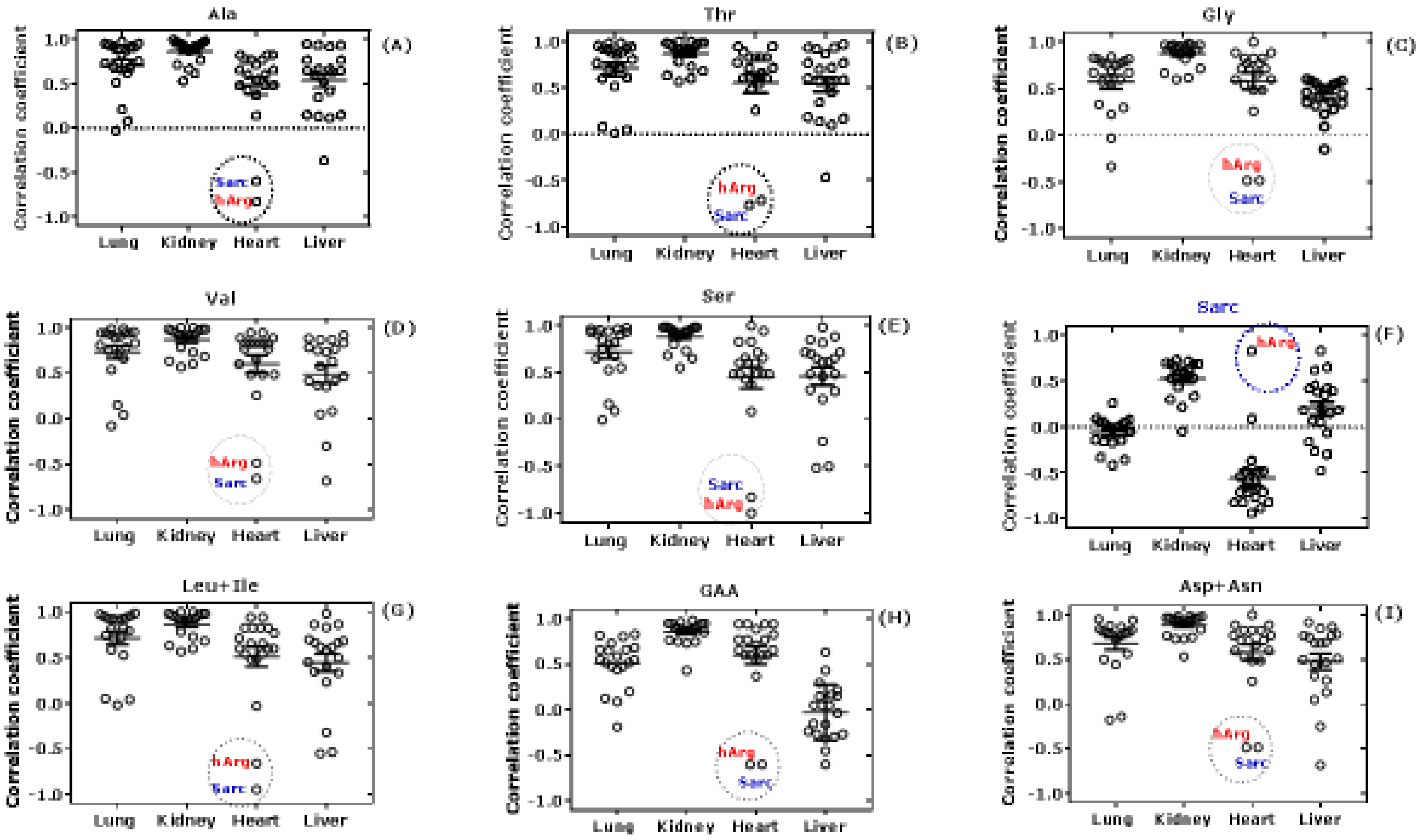
Ala
(A)
Thr
(B)
Gly
(C)
Val
(D)
Ser
(E)
Sarc
(F)
Leu+Ile
(G)
GAA
(H)
Asp+Asn
(I)
Correlation coefficient
1.0
0.5
0.0
-0.5
-1.0
Lung
Kidney
Heart
Liver
Sarc
hArg

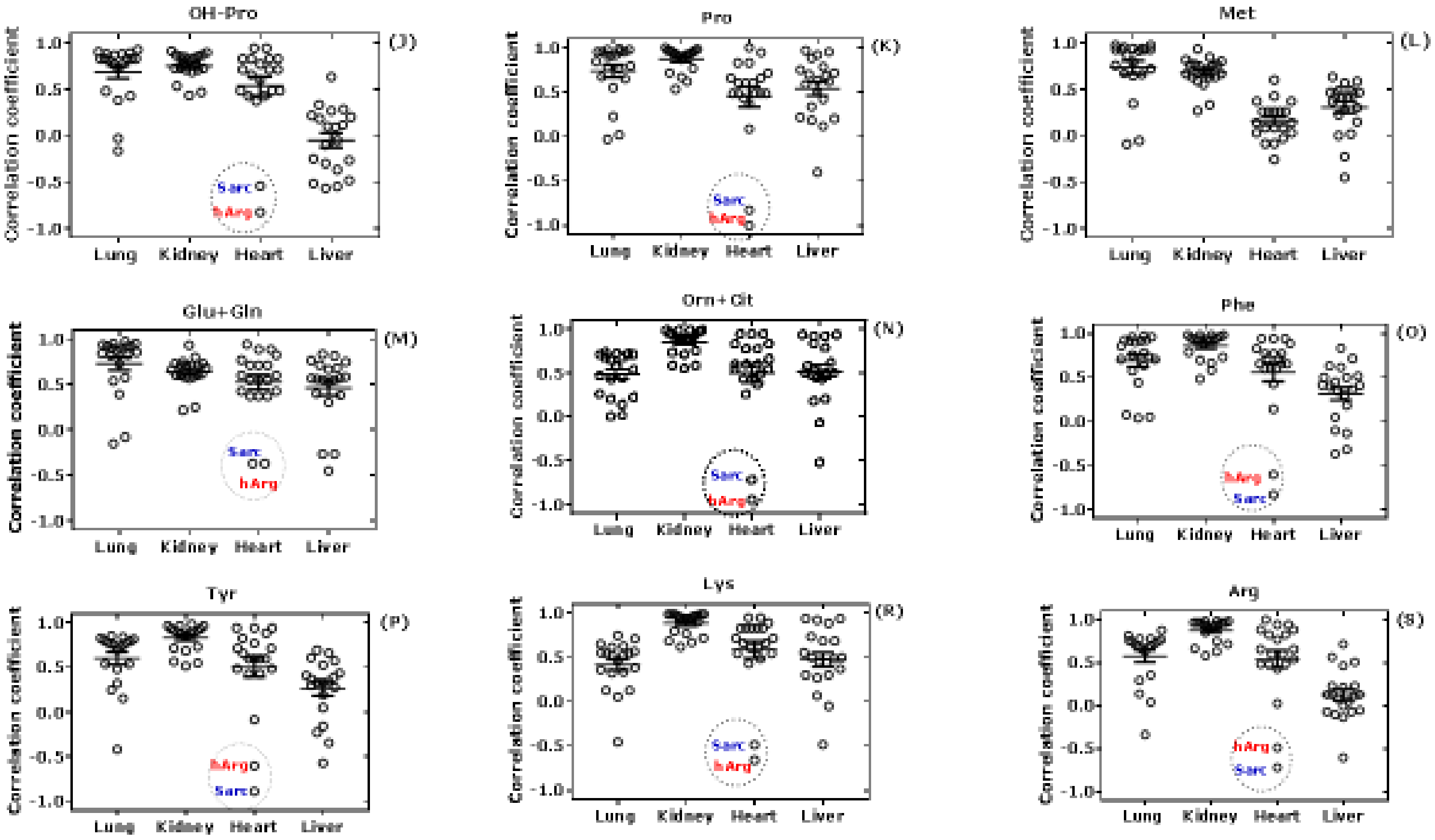
OH-Pro
Pro
Met
Glu+Gln
Orn+Cit
Phe
Tyr
Lys
Arg
(J)
(K)
(L)
(M)
(N)
(O)
(P)
(R)
(S)
Correlation coefficient
1.0
0.5
0.0
-0.5
-1.0
Lung
Kidney
Heart
Liver
Sarc
hArg

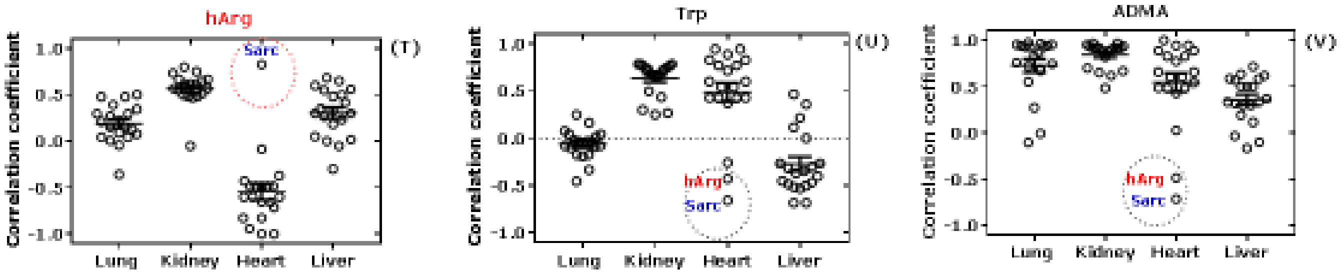

Figure S2. Spearman correlation coefficients for free tissue amino acids in lung, kidney, heart and liver of the study rats. The data points for Sarc and hArg are encircled and given in blue and red, respectively. Explanation of the amino acids: Ala, alanine; Thr, threonine; Gly, Glycine; Val, Valine; Ser, Serine; Sarc, sarcosine; Leu+Ile, leucine+isoleucine; GAA, Guanidinoacetate; Asp+Asn, aspartate+asparagine; OH-hydroxyproline; Pro, proline; Met, methionine; Glu+Gln, glutamate+glutamine; Orn+Cit, ornithine+citrulline; Phe, phenylalanine; Tyr, tyrosine; Lys, lysine; Arg, arginine; hArg, homoarginine; Trp, tryptophane; ADMA, asymmetric dimethylarginine.

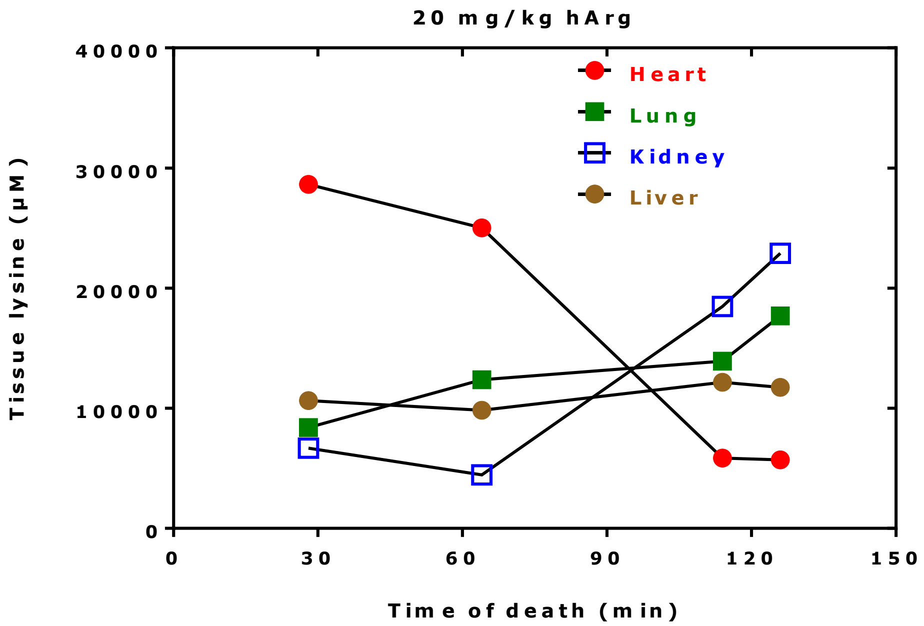

Figure S3. Concentration of free Lys in homogenate samples of four rats (#3, #2, #18, #13) administered with hArg (t=0 min) and isoprenaline (t = 15 min) measured at the time of death. See also Table S1.
